# Phylogenetic diversity and functional potential of the microbial communities along the Bay of Bengal coast

**DOI:** 10.1038/s41598-023-43306-4

**Published:** 2023-09-25

**Authors:** Salma Akter, M. Shaminur Rahman, Hazrat Ali, Benjamin Minch, Kaniz Mehzabin, Md. Moradul Siddique, Syed Md. Galib, Farida Yesmin, Nafisa Azmuda, Nihad Adnan, Nur A. Hasan, Sabita Rezwana Rahman, Mohammad Moniruzzaman, Md Firoz Ahmed

**Affiliations:** 1 Department of Microbiology, Jahangirnagar University, Savar, Dhaka, Bangladesh; 2 Department of Microbiology, Jashore University of Science and Technology, Jashore, Bangladesh; 3 Department of Marine Biology and Ecology, Rosenstiel School of Marine, Atmospheric, and Earth Science, University of Miami, Coral Gables, FL USA; 4 Department of Computer Science and Engineering, Jashore University of Science and Technology, Jashore, Bangladesh; 5 University of Maryland, College Park, MD USA; 6 Department of Microbiology, University of Dhaka, Dhaka, Bangladesh

**Keywords:** Data publication and archiving, Microbial communities, Environmental microbiology

## Abstract

The Bay of Bengal, the world's largest bay, is bordered by populous countries and rich in resources like fisheries, oil, gas, and minerals, while also hosting diverse marine ecosystems such as coral reefs, mangroves, and seagrass beds; regrettably, its microbial diversity and ecological significance have received limited research attention. Here, we present amplicon (16S and 18S) profiling and shotgun metagenomics data regarding microbial communities from BoB’s eastern coast, viz., Saint Martin and Cox’s Bazar, Bangladesh. From the 16S barcoding data, Proteobacteria appeared to be the dominant phylum in both locations, with *Alteromonas*, *Methylophaga*, *Anaerospora*, *Marivita*, and *Vibrio* dominating in Cox’s Bazar and *Pseudoalteromonas*, *Nautella*, *Marinomonas*, *Vibrio*, and *Alteromonas* dominating the Saint Martin site. From the 18S barcoding data, Ochrophyta, Chlorophyta, and Protalveolata appeared among the most abundant eukaryotic divisions in both locations, with significantly higher abundance of Choanoflagellida, Florideophycidae, and Dinoflagellata in Cox’s Bazar. The shotgun sequencing data reveals that in both locations, *Alteromonas* is the most prevalent bacterial genus, closely paralleling the dominance observed in the metabarcoding data, with *Methylophaga* in Cox’s Bazar and *Vibrio* in Saint Martin. Functional annotations revealed that the microbial communities in these samples harbor genes for biofilm formation, quorum sensing, xenobiotics degradation, antimicrobial resistance, and a variety of other processes. Together, these results provide the first molecular insight into the functional and phylogenetic diversity of microbes along the BoB coast of Bangladesh. This baseline understanding of microbial community structure and functional potential will be critical for assessing impacts of climate change, pollution, and other anthropogenic disturbances on this ecologically and economically vital bay.

## Introduction

The oceans cover 70% of the earth's surface and are home a myriad of microorganisms, all of which contribute to the survival of life on earth^1^. These microorganisms are important for the health of aquatic ecosystems that vary geographically due to environmental conditions, community adaptability, and anthropogenic impacts^2,3^. Global change is expected to influence both the mean and variance of environmental parameters in the open sea, with global pH decreases and ocean surface water temperature rises^4,5^. As microbes play a significant role in marine nutrient cycling, climate models should account for changes in microbial community structure and biogeochemical activities^6–8^.

The coastline of the Bengal delta comprises the Bay of Bengal (BoB), the largest bay in the world^9^. Due to considerable influence by seasonal natural disasters such as monsoon rainfalls, climate disasters, and human development, the BoB gets a significant flux of fresh and cold river water into this semi-enclosed tropical ocean basin in the northeast Indian Ocean^9,10^. Rising surface water temperatures in the BoB have led to heightened stratification in the water column, creating zones characterized by depleted oxygen and nutrient levels^10^.

The coastal ecosystem provides vast scope for economic development through the establishment of ports, fisheries industries, gas fields, oil refineries, and naval stations. Despite enormous economic contributions to coastal countries like Bangladesh, India, Myanmar, and Sri Lanka, the BoB ecosystem is extremely underexplored. Several reports from neighboring countries showed investigative outcomes on oceanography, phytoplanktonic diversity, and stratification-induced nutrient cycling, but without a notable focus on microbial composition through advanced molecular studies^9,11–13^.

Multiple studies have found that BoB oceanic characteristics have a significant impact on the composition and metabolic diversity of the marine microbiome^14,15^. Recent large-scale projects in conjunction with modern DNA sequencing technologies have made significant contributions to the microbial characterization of numerous marine ecosystems, ranging from the Arctic Ocean to the tropics^16–19^. Several studies have reported the microbial diversity of the surface and sub-surface regions of BoB^13,20,21^, but no comprehensive study has been performed yet on this important ecological system. These coastal regions of Bangladesh play an important economic role because they are the most visited tourist destination in the country^22,23^ and the largest source of fisheries-based rural markets, supplying a significant portion of the country's fish^24^.

This study aims to address this knowledge gap by utilizing high-throughput 16S, 18S, and metagenomic sequencing to identify prokaryotic and eukaryotic microbial diversity in two distinct coastal regions of Bangladesh. We seek to provide critical insights into the composition, metabolic diversity, prevalence of pathogens, and antimicrobial resistance markers in these economically and ecologically vital ecosystems^25^. The findings will expand our fundamental understanding of coastal marine microbiomes while informing environmental conservation and public health efforts in the region.

## Methodology

### Sample collection

The seawater samples were collected in duplicates from two distinct coastal regions of Bangladesh: Cox’s Bazar and the Saint Martin. The sampling was done on March 2 and 3, 2022 during low tide. The samples were collected in 1 L sterile sampling bottles at 1.5-m depth from the surface water. The bottles were sealed underwater and transported to the MicrobiOmics and Translational Research Laboratory at Department of Microbiology, Jahangirnagar University, Bangladesh for further processing. Insulated plastic boxes were used for sample transportation to maintain the cold-chain and samples were reached within 24 h of collection. The samples from Saint Martin were labeled as S5, S6, S7 and S8 and the samples from Cox’s Bazar were labeled as S9, S10, S11 and S12. The geographical location of each sampling sites is available in Supplementary Data-1 (Metadata). Water samples from each site were taken in sterile beaker and physicochemical parameters like temperature, pH, salinity, and total dissolved solids (TDS) were measured on-site using physicochemical meter (Hanna, USA).

### Total DNA extraction from water samples/molecular processing

The water samples were initially passed through Whatman filter Paper No. 1 (pore size 11 µm) to get rid of any large debris. The water filtrates were then filtered through the Millipore filtration unit, firstly through 0.45 µm membrane and subsequently through 0.20 µm membrane. The filtrate was discarded, and the filter papers were folded in 5 ml sterile tubes and stored at − 80 °C for DNA extraction. Total DNA was extracted from the filter papers using DNeasy PowerWater Kit (Qiagen, USA) according to the manufacturer's protocol. The purified DNA extracted from duplicates samples of a single site were combined together and were quantified using a NanoDrop2000 (Thermo Scientific, USA) to determine concentration and relative purities by A_260/280_ ratio, prior to sending for 16S and 18S rDNA based metabarcoding done by EzBiome, USA. For whole genome metagenomic (shotgun) sequencing, equal quantity of the extracted DNA from both 0.45 and 0.22 µm membranes from representative four sampling sites of two locations were combined as pooled samples (Cox’s Bazar (S2) and Saint Martin (S1)).

### Library preparation and sequencing

The amplification of prokaryotic DNA was achieved by targeting the V3–V4 region of 16S rRNA gene with 30 μL final volume containing 15 μL of 2X master mix (BioLabs, USA), 3 μL of template DNA, 1.5 μL of each V3–V4 forward and reverse primers, 341F (5′-CCT ACG GGNGGCWGCAG-3′) and 806R (5′-GACTACHVGGGTATCTAATCC-3′), respectively^26^. For the remaining volume, 9 μL of DEPC treated ddH_2_O was added. A 25 cycle of PCR amplification including initial denaturation at 95 °C for 3 min, denaturation at 95 °C for 30 s, primer annealing at 55 °C for 30 s and elongation at 72 °C for 30 s was performed for bacterial DNA with the final extension of 5 min at 7 °C in a thermal cycler (Analytik Jena, Germany).

To amplify 18S DNA, the universal eukaryotic primers set 1391F (5ʹ-GTA CAC ACC GCC CGTC-3ʹ) / EukBr (5ʹ- TGA TCC TTC TGC AGG TTC ACC TAC-3ʹ) spanning the V9 region of 18S rRNA gene were utilized^26^. PCR mixture for the amplification of fungal DNA was the same as the one used for prokaryotic DNA. For eukaryotic DNA, a thirty-five cycles of PCR amplification were run with the temperature profile of initial denaturation at 94 °C for 3 min, denaturation at 94 °C for 45 s, annealing at 57 °C for 1 min, elongation at 72 °C for 1.5 min and final extension of 10 min at 72 °C. After electrophoresis, the PCR amplicons were visualized in 1.5% agarose gel prepared in 1X TAE buffer. Agencourt Ampure XP beads (Beckman Coulter, Brea, USA) were used for PCR products purification, and the Nextera XT index kit (Illumina, San Diego, USA) for paired-end library preparation according to Illumina standard protocol (Part# 15,044,223 Rev. B). Followed by normalizing the DNA concentration for all samples according the Standard Illumina Protocol, paired-end (2 × 300 bp reads) sequencing of the prepared library pools was performed using MiSeq high throughput kit (v3 kit, 600 cycles) with an Illumina MiSeq platform (Illumina, USA)^27,28^.

### Bioinformatics data processing

The generated FASTQ files were evaluated for quality using FastQC v0.11^29^. Adapter sequences, and low-quality ends per read were trimmed by using Trimmomatic v0.39 with a sliding window size of 4; a minimum average quality score of 20; minimum read length of 40 bp^30^. After quality control, there were an average of 9305 pairs of reads for 16S samples (minimum = 7476 and maximum = 11,961 pairs) and an average of 34,144 pairs of reads for 18S samples (minimum = 51,681 and maximum = 22,392 pairs). QIIME2 (2022.2), an integrated pipeline was used for OTU clustering, phylogenetic estimation and taxonomic assignment^31^. VSEARCH metagenomics algorithm integrated in QIIME2 was employed for read joining, dereplicate-sequences, de novo clustering (OTU clustering with 99% identity), de novo chimera checking (exclude chimeras and “borderline chimeras”)^32^. To generate a tree for phylogenetic diversity analyses, MAFFT^33^ was used for alignment and FastTree (v2.1.8) was used to build the tree^34^.

For taxonomic assignment, Greengenes (v13_5) database (99% OTU and taxonomy) used for prokaryotic taxonomic assignment (16S) and SILVA(v132_99) database (99% OTU and taxonomy) were also used for eukaryotic taxonomic assignment^35,36^. The reference database was trained using the 16S and 18S sequencing primer pairs using a Naive-Bayes classifier^37,38^. Classify-sklearn algorithms were utilized to classify the assigned OTU for prokaryotic and eukaryotic samples^39,40^.

### Statistical analysis

The downstream analysis, which included alpha and beta diversity, microbiological composition, and statistical comparison, were performed using the Phyloseq (version 4.2) package^41,42^ for R(v 4.2.1)^43,44^. The OTU data were normalized by the total sum scaling techniques (TSS) included in the Phyloseq R package. Observed, Chao1, Shannon, Simpson, InvSimpson, and Fisher alpha diversity were estimated and plotted by using “Vegan”, “ggplot2”, and “ggpubr” R packages. The Wilcoxon sum rank test in the “microbiomeutilities” R package (https://microsud.github.io/microbiomeutilities/) was used to evaluate the differences in microbial diversity and abundance between two locations. Beta diversity was measured with the principal coordinate analysis (PCoA) using Bray–Curtis, weighted unifrac, and unweighted unifrac dissimilarity matrices, and permutational multivariate analysis of variance (PERMANOVA) with 999 permutations was used to estimate a *p* value for differences between two locations. The non-metric multidimensional scaling (NMDS) method was also applied for the above-mentioned distance metrics including PERMANOVA. Phyloseq, Vegan, microbiome utilities, and ggplot2 packages were employed for taxonomic comparison and plotting^41,45–48^. To analyze and illustrate the data, the R packages Hmisc and corrplot were used^49–51^.

### Shotgun metagenomic sequencing, and sequence reads preprocessing

Both Cox's Bazar and Saint Martin's samples were combined into two different pools before submission to shotgun metagenomic sequencing. Shotgun metagenomic (WMS) libraries were prepared with Nextera XT DNA Library Preparation Kit and paired-end (2 × 150 bp) sequencing was performed on a NovaSeq 6000 sequencer (Illumina Inc., USA) from EzBiome, USA. The generated FASTQ files were evaluated for quality using FastQC v0.11^29^. Adapter sequences, and low-quality ends per read were trimmed by using Trimmomatic v0.39 with a sliding window size of 4; a minimum average quality score of 20; minimum read length of 50 bp^30^. In the end, the trimmed read counts for S1 and S2 were 33.94 and 31.8 million, or 92.20 and 92.37% of the total raw read counts, respectively.

### Taxonomic mapping, classification, and phylogenetics study

CZID (previously IDseq), a real time microbiome characterization pipeline (v7.1)^52^ and EzBioCloud taxonomic profiling^53^ were used for taxonomic identification of the short read sequences. CZID is an open-source cloud-based pipeline for taxonomic assignments against the NCBI non-redundant (NR) database with NRL (NRL; non-redundant nucleotide alignment length in bp) ≥ 50 and NR % identity ≥ 80. CZID applies host filtering, alignment with minimap2^54^ assembly with SPAdes^55^ and blast for taxonomic assignment.

Bacteria, Archaea, Virus and cdf (https://www.ncbi.nlm.nih.gov/refseq/) were also added to the Kraken2 database^56^. After acquiring a list of candidate species, a custom bowtie2^57,58^ database was built utilizing the core genes and genomes from the species found during the first step. The raw sample was then mapped against the bowtie2 database using the very sensitive option and a quality threshold of phred20. Samtools^59,60^ was used to convert and sort the output BAM file. Coverage of the mapped reads against the bam file was obtained using Bedtools^61,62^. Then, to avoid false positives, using an in-house script, we quantified all the reads that mapped to a given species only if the total coverage of their core genes (archaea, bacteria) or genome (fungi, virus) was at least 25%. Finally, species abundance was calculated using the total number of reads counted and normalized species abundance was calculated by using the total length of all their references.

### Shotgun sequence assembly

Short reads from both metagenomic libraries were quality trimmed using Trim Galore (https://github.com/FelixKrueger/TrimGalore) with default parameters^63^. The trimmed data was assembled using metaSPADES^64–66^ with default parameters and a minimum contig size of 1500 base pairs. Gene prediction of the metagenomic contigs was done using Prodigal^67^ with the meta option.

### Functional profiling and BRITE hierarchy analysis

For each sample, functional annotations were obtained by matching each read against the KEGG database using DIAMOND^54,68–70^. DIAMOND was executed using the blastx parameter, which converts each metagenomic read into multiple amino acid sequences by generating all six open reading frame variations, and then matches it against the pre-built KEGG database. After quantifying all the KEGG orthologs present, minpath^71^ was used to predict the presence of KEGG functional pathways. The KEGG BRITE database is a collection of BRITE hierarchy files, called htext (hierarchical text) files, with additional files for binary relations. The htext file is manually created with in-house software called KegHierEditor^72^. The htext file contains “A”, “B”, “C”, etc. at the first column to indicate the hierarchy level, and may contain multiple tab-delimited columns. Thus, the htext file is like an Excel file with the additional first field for the hierarchy level. The BRITE hierarchy file has been created to represent the functional hierarchy of KEGG objects identified by the KEGG Identifiers.

### Profiling of antimicrobial resistance genes (ARGs), mobile genetic elements and virulence factor-associated Genes (VFGs)

For antimicrobial resistance profiling, two different pipelines were used. The first is the AMR++pipeline with the Microbial Ecology Group (MEG) antimicrobial resistance database (MEGARes v3.0.0)^73–75^. The short reads were aligned to the MEGARes database using Burrows-Wheeler Aligner (BWA)^76^, with the gene fraction (the percentage of genes that were matched to by at least one sequencing read) set to ≥ 80%. Contigs obtained from CZID pipeline and refined bins were also aligned against the MEGARes database with ≥ 80% identity and ≥ 80% subject coverage. In addition, the EzBioCloud pipeline was also used to assign ARGs from short reads. Antibiotic resistance gene profiles were produced by using a pre-built bowtie2^57^ database composed of NCBI’s National Database of Antibiotic Resistant Organisms (NDARO, (www.ncbi.nlm.nih.gov/pathogens/antimicrobial-resistance/) reference genes. Each read of the metagenome sample was mapped against these genes using bowtie2 with the very-sensitive option, and the output was then converted and sorted by Samtools^60^. Finally, for each gene found, depth and coverage were calculated by using Samtool’s mpileup script. We used the same pipelines mentioned above to find virulence factor-associated genes (VFGs) from the Virulence Factors of Pathogenic Bacteria (VFDB) database^74,77–79^. Insertion Sequence (IS) were determined by ISEScan (v1.7.2.3) (https://github.com/xiezhq/ISEScan) ISEScan is a software pipeline that is based on profile hidden Markov models derived from carefully curated IS components. It is implemented in Python and is known for its high sensitivity^80^. Prokaryotic transposons were identified by BLAST search against TnCentral (https://tncentral.ncc.unesp.br/index.html) prokaryotic transposon database with ≥ 90 percent identities^81^. Refined assembled files were used for both ISEScan and TnCentral database search. For the purpose of conducting a plasmid sequence search, the software tool plaSquid (available at https://github.com/mgimenez720/plaSquid) was employed. plaSquid is specifically designed for identifying plasmid sequences within metagenomic assemblies, and the default settings of the tool were used in this study^82^.

## Results

### Physicochemical properties of water samples

**Figure 1:**
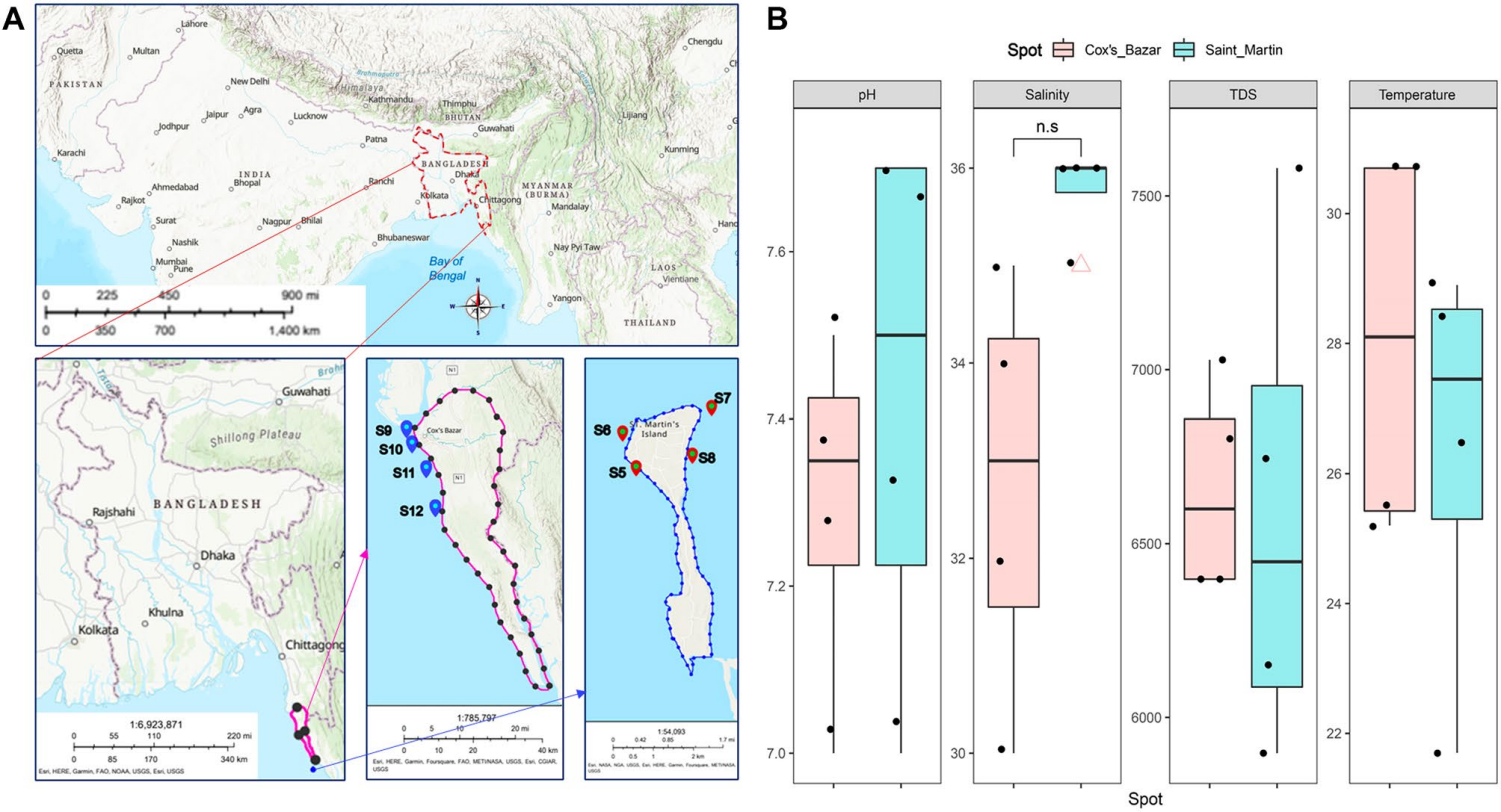
Sampling location and their physicochemical properties. (**A**) Two sampling locations (Cox’s Bazar and Saint Martin) are indicated. (**B**) The physicochemical parameters (pH, salinity, TDS and Temperature) of each are plotted on boxplots and comparisons were made with t-test. The map was constructed using ArcGIS online platform.

A total of 8(= n) samples were collected from the coastal regions of Cox’s bazar (n_c_ = 4) and Saint Martin (n_s_ = 4) Bangladesh during the 2nd and 3rd of March 2022. (Fig. 1A). The samples from Cox's Bazar had an average pH of 7.3, while the ones from Saint Martin's had a slightly higher average pH of 7.425. The maximum salinity, TDS, and temperature in samples from Cox’s Bazar were, 35 units (average = 32.75), 7028 units (average = 6656.5), and 30.7 °C (average = 28.03 °C) respectively, and in samples from Saint Martin were 36 units (mean = 35.75), 7580 units (mean = 6593.25), and 30.7 °C (mean = 28.03 °C) respectively (Fig. 1B). No statistically significant variations have been observed in the physicochemical parameters among samples from these two locations (t-test, *p* > 0.05) (Fig. 1B) (Supplementary Data 1: Metadata).

### 16S and 18S Microbiome diversity

#### Bacterial and Archaeal Diversity from 16S amplicons

**Figure 2:**
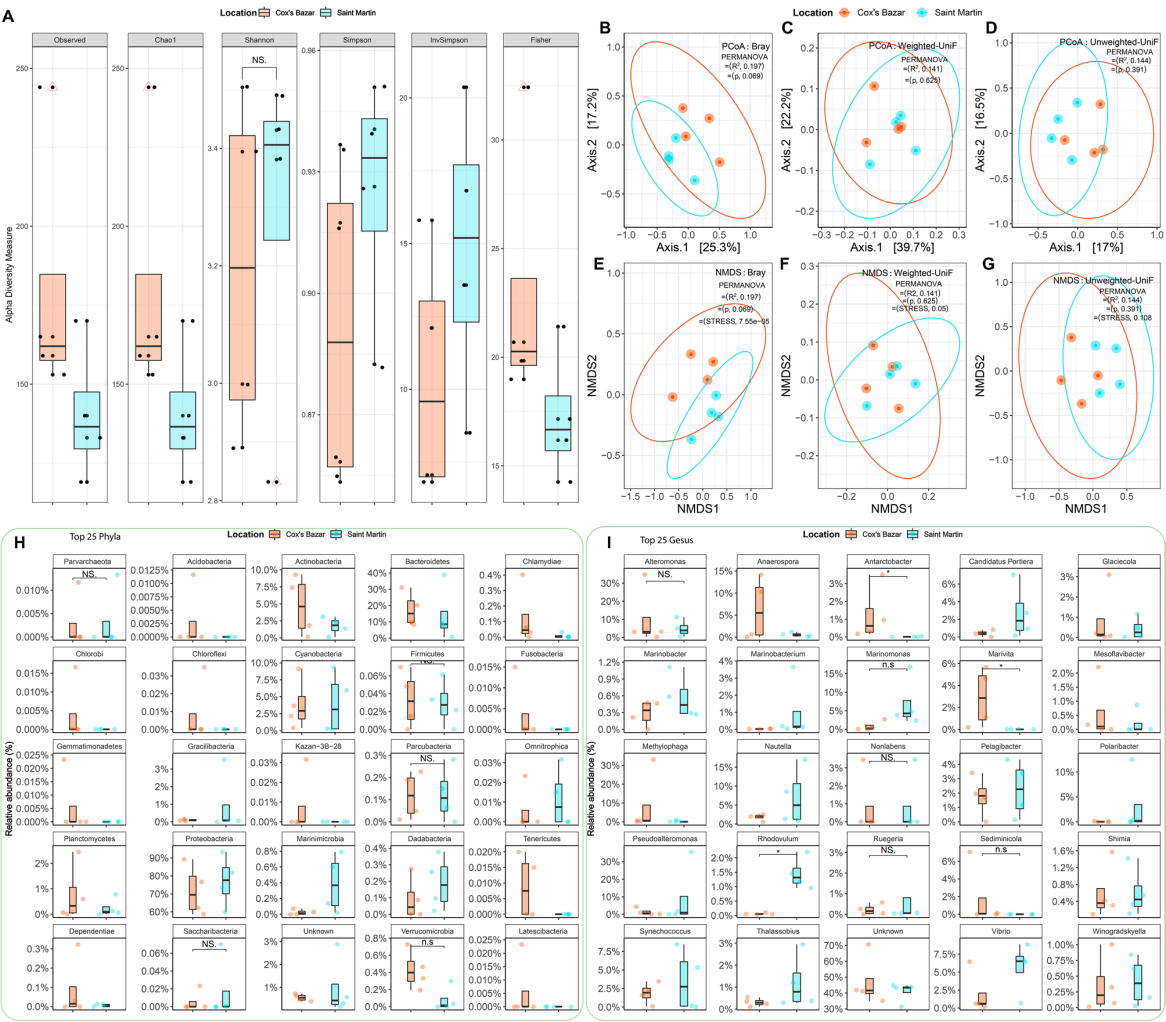
Bacterial and Archaeal alpha- and beta-diversity and taxonomic abundance based on 16S amplicon sequencing data. (**A**) For the prokaryotic (bacteria and archaea) microbial community of Cox's Bazar and Saint Martin samples, the observed species, Chao1, Shannon, Simpson, InvSimpson, and Fisher diversity (Alpha diversity) indices were estimated. X-axis represents the location and y-axis represents the alpha diversity measure. The diversity for each is plotted using boxplots, and the pairwise Wilcoxon sum rank test is used to compare them. (**B**–**G**) Beta diversity measures of the prokaryotic (bacteria and archaea) microbial community. Principal coordinate analysis (PCoA) (**B**–**D**) and non-metric multidimensional scaling (**E**–**G**) were performed using Bray, Weighted-Unifrac, and Unweighted-Unifrac distance metrics for the two locations of samples. Permutational multivariate analysis of variance (PERMANOVA) was performed with 999 permutations to estimate a significance (*p* value) for differences between two locations. PERMANOVA with 999 permutations was used to determine the significance (*p* value) of differences between two locations. Significance level (*p* value) 0.0001, 0.001, 0.01, 0.05, and 0.1 are represented by the symbols "****", "***", "**", "*", and “n.s”, respectively. Stress value represents the goodness of fit of NMDS (> 0.2 Poor, 0.1–0.2, Fair, 0.05–0.1 Good, and < 0.05 Excellent). (**H**) Comparison of relative abundance of twenty-five prokaryotic phyla and (**I**) Genus in the two different locations (Cox’s Bazar and Saint Martin). The diversity for each division is plotted on boxplots and comparisons are made with Wilcoxon sum rank test. Significance level (*p* value) 0.0001, 0.001, 0.01, 0.05, and 0.1 are represented by the symbols “****”, “***”, “**”, “*”, and “n.s”, respectively.

We were able to get a total of 397 OTUs (Operational Taxonomic Units) from the 16S microbiome sequences derived from V3–V4 amplicons of all the samples. After clustering and filtering for chimeras, the Observed, Chao1, Shannon, Simpson, InvSimpson, and Fisher indices were examined for within-sample diversity (Alpha diversity), but the results showed that there was no significant difference (Wilcoxon signed-rank test, *p* > 0.05) between the two locations in terms of bacterial and archaeal diversity (Fig. 2A). Principal coordinate analysis (PCoA) with Bray Curtis distance (Fig. 2B), weighted unifrac distance (Fig. 2C), and unweighted unifrac distance (Fig. 2D) showed that there were no significant differences between the two sampling locations (beta diversity) (PERMANOVA, *p* > 0.05). Similar results were obtained using the non-metric multidimensional scaling (NMDS) technique, with no discernible differences (PERMANOVA, *p* > 0.05) (Fig. 2E–G).

Our study revealed the presence of a total of 24 bacterial phyla and one archaeal phylum (Parvarachaeota) in the sequence data (Fig. 2H). 16 bacterial phyla were found in the Saint Martin region, in contrast to the 24 that were found in Cox's Bazar (Supplementary Figure-1A). All 16 phyla that were found in Saint Martin were also found in Cox's Bazar. More than 98% of the bacterial phyla in the Cox's Bazar area were comprised of Proteobacteria (71.7%), Bacteroidetes (17.4%), Actinobacteria (4.7%), Cyanobacteria (3.8%), and Planctomycetes (0.8%). On the other hand, almost 97% of all phyla in the Saint Martin were Proteobacteria (77.1%), Bacteroidetes (14.2%), Cyanobacteria (3.95%), and Actinobacteria (1.7%) (Fig. 2H).

From the QIIME2 analysis of 397 OTUs, a total of 133 bacterial genera were identified cumulatively from both locations, among them 70 genera were commonly present in both S1 and S2 samples (Supplementary Data-1). Interestingly, 18 genera have been found in 16S datasets from S5–S8 samples, whereas 45 genera have found in datasets from S9–S12 samples (Supplementary Data-1). Notably, the top ten genera from Cox’s Bazar had 84.4% relative abundance, consisting of sequences that could not be assigned to any known phyla (47.21%), *Alteromonas* (10.27%), *Methylophaga* (8.57%), *Anaerospora* (6.31%), *Marivita* (2.89%), *Vibrio* (1.97%), *Synechococcus* (1.85%), *Sediminicola* (1.79%), *Nautella* (1.78%), and *Pelagibacter* (1.75%) (Fig. 2I). On the contrary, the top ten genera from Saint Martin had 94.2% relative abundance, consisting of sequences with unknown assignment (40.72%), *Pseudoalteromonas* (9.39%), *Nautella* (6.96%), *Marinomonas* (6.92%), *Vibrio* (5.64%), *Alteromonas* (4.85%), *Synechococcus* (3.49%), *Polaribacter* (3.23%), *Candidatus Portiera* (2.72%) and *Pelagibacter* (2.26%) (Supplementary Data 1). Considering the genus level, Cox’s Bazar had significantly higher abundance for *Antarctobacter* (Wilcoxon rank test *p* value = 0.029), *Formosa* (*p* value = 0.029) and *Marivita* (*p* value = 0.021) and Saint Martin had significantly higher abundance for *Oleibacter* (*p* value = 0.029), and *Rhodovulum* (*p* value = 0.029) (Supplementary Figure-2).

#### Diversity of microbial eukaryotes from 18S amplicons

**Figure 3:**
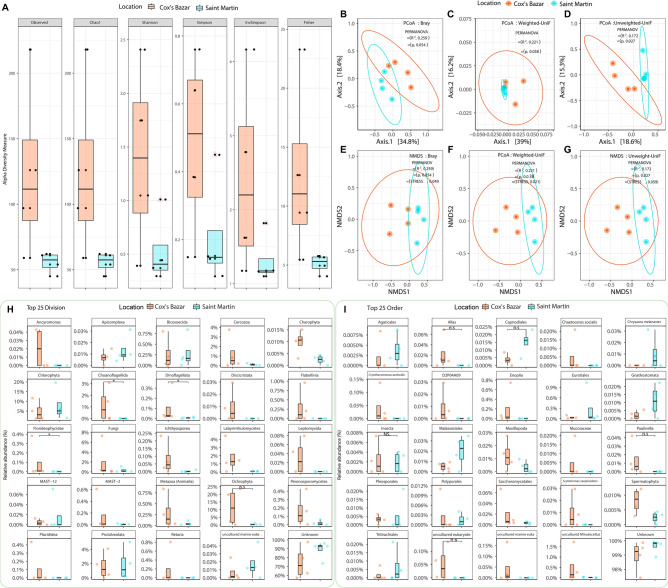
Eukaryotic microbial diversity and taxonomic abundance based on 18S amplicon sequencing data. (**A**) The observed species, Chao1, Shannon, Simpson, InvSimpson, and Fisher diversity (Alpha diversity) measures were used to estimate the Eukaryotic microbial community diversity of Cox's Bazar and Saint Martin samples as described for the prokaryotic microbes. (**B**–**G**) Beta diversity of the eukaryotic microbial community was estimated here as described in Fig. 2B–G. For the two sample sites, Bray, Weighted-Unifrac, and Unweighted-Unifrac distance measures were used. Permutational multivariate analysis of variance (PERMANOVA) was performed with 999 permutations to estimate a significance (*p* value) for differences between two locations. PERMANOVA with 999 permutations was used to determine the significance (*p* value) of differences between two locations. Significance level (*p* value) 0.0001, 0.001, 0.01, 0.05, and 0.1 are represented by the symbols "****", "***", "**", "*", and "n.s", respectively. Stress value represents the goodness of fit of NMDS (> 0.2 Poor, 0.1–0.2, Fair, 0.05–0.1 Good, and < 0.05 Excellent). (**H**) Comparison of relative abundance of twenty-five eukaryotic divisions and (**I**) Order in the two different locations (Cox’s Bazar and Saint Martin). The diversity for each order is plotted and differences were tested using Wilcoxon sum rank test. Significance level (*p* value) 0.0001, 0.001, 0.01, 0.05, and 0.1 are represented by the symbols “****”, “***”, “**”, “*”, and “n.s”, respectively.

After clustering and screening for chimeras from the V9-amplicons of 18S microbiome sequencing, we were able to get a total of 693 OTUs (Operational Taxonomic Units) from all samples (S5–S12) (Supplementary Data-1). Observed, Chao1, Shannon, Simpson, InvSimpson, and Fisher indices no significant difference within sample (alpha) diversity (Wilcoxon signed-rank test, *p* > 0.05) between the Cox's Bazar and Saint Martin’s samples. (Fig. 3A). Principal coordinate analysis (PCoA) using Bray Curtis distance (Fig. 3B), weighted unifrac distance (Fig. 3C), and unweighted unifrac distance (Fig. 3D) revealed considerable differences (PERMANOVA, *p* < 0.05) between the two sampling locations of samples. An NMDS approach revealed the same significant difference (PERMANOVA, *p* < 0.05) (Fig. 3E–G). A closer examination revealed that three and nineteen divisions were unique to Saint Martin and Cox's Bazar, respectively, with the remaining 22 divisions shared by both locations (Supplementary Figure-1B). In both Cox's Bazar and Saint Martin, a large proportion of the OTUs could not be assigned to known divisions (74.27% and 88.85% respectively). In Cox's Bazar, the most abundant divisions found are Ochrophyta (11.44%), Chlorophyta (4.72%), Fungi (1.98%), Labyrinthulomycetes (1.76%), Protalveolata (1.61%), Cercozoa (1.19%), and Choanoflagellida (1.15%). In the Saint Martin samples, Chlorophyta (7.77%) was the most abundant flowed by Protalveolata (1.88%), Ochrophyta (0.49%) and Fungi (0.37%) (Supplementary Data-1). Between the two sites, only Choanoflagellida (*p* = 0.021), Florideophycidae (*p* = 0.021), and Dinoflagellata (*p* = 0.029) were found to have significantly different abundance, all being higher in Cox’s bazar (Fig. 3H). Likewise, a significant proportion of order among the top 25, has been identified as uncultured marine eukaryote after deducing the top 25 order determined in 18S dataset (Fig. 3I). Interestingly, the diversity of unidentified marine eukaryotic order is higher in samples from Cox’s bazar (S9–S12), indicating the prevalence of yet-to-identify eukaryotic population in microbiome of BoB. Besides, the highly diverse and abundant eukaryotic orders prevalent in samples from Saint Martin are Agaricales, Chrysaora, Gnathostomata, Malasseziales and Tritirachiales (Fig. 3I).

#### Site specific relative abundance of different genera

**Figure 4:**
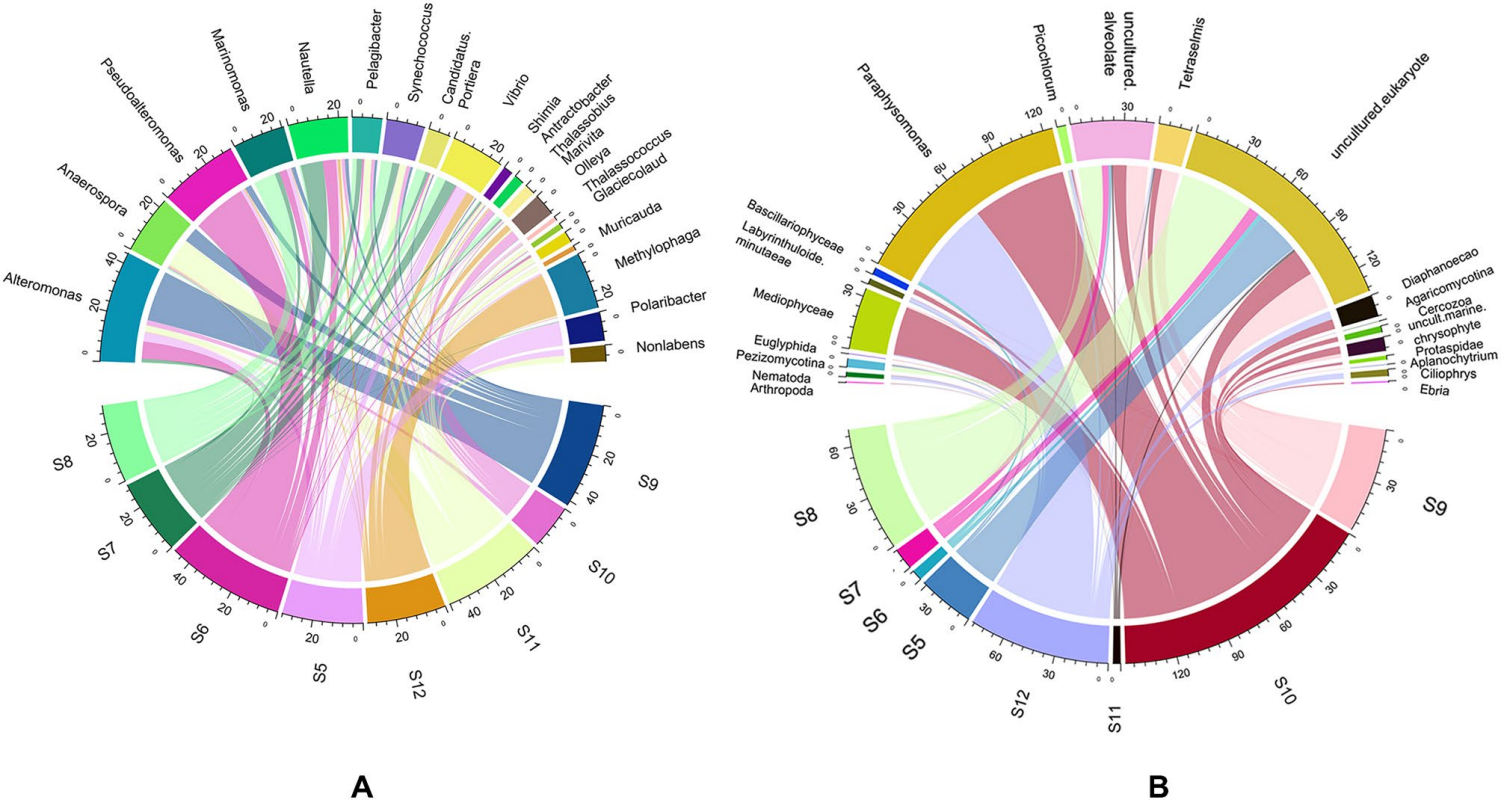
Circos representation of relative abundance for the top 20 prokaryotic genera (**A**) from 16S rRNA sequence data and top 20 eukaryotic genera (**B**) from 18S rRNA rRNA sequence data obtained across different sampling sites. Sample S5-S8 belong to Saint Martin and S9–S12 belong to Cox’s Bazar. The representing values are the 1st percentile of the actual read numbers.

The relative abundance of the dominant genera in the samples of eight sites showed significant variations in the dominance of bacterial genus (Fig. 4A). Among the top 20 genera, *Alteromonas* appeared to be the most dominant one with highest abundance in S9 sample, followed by *Pseudoalteromonas* which was most abundant in S6. The next abundant genera, *Anaerospora,* was dominant in S11. Among the other genus *Methylophaga* and *Polaribacter* mostly belonged to S12 and S5 respectively. Other genera like *Vibrio* and *Nautella* were distributed in all the samples.

The 18S sequence data showed the maximum relative abundance read for S10 and that was followed by S12, S8 and S9 (Fig. 4B). Among sites, the majority of taxa remained unknown. *Paraphysomonas* was the most abundant genera and was almost equally distributed to S10 and S12 sites. *Mediophyceae*, the next dominant eukaryotic genera were found exclusively in S10. Another most abundant taxa, uncultured alveolates, was mostly associated to S9 and S8 however, but were also present in other samples. Overall, the differences in relative abundance for the top 20 genera was more noticeable for the eukaryotic organisms than prokaryotic ones in the sampling sites.

### Impact of environmental conditions on microbial community composition

**Figure 5:**
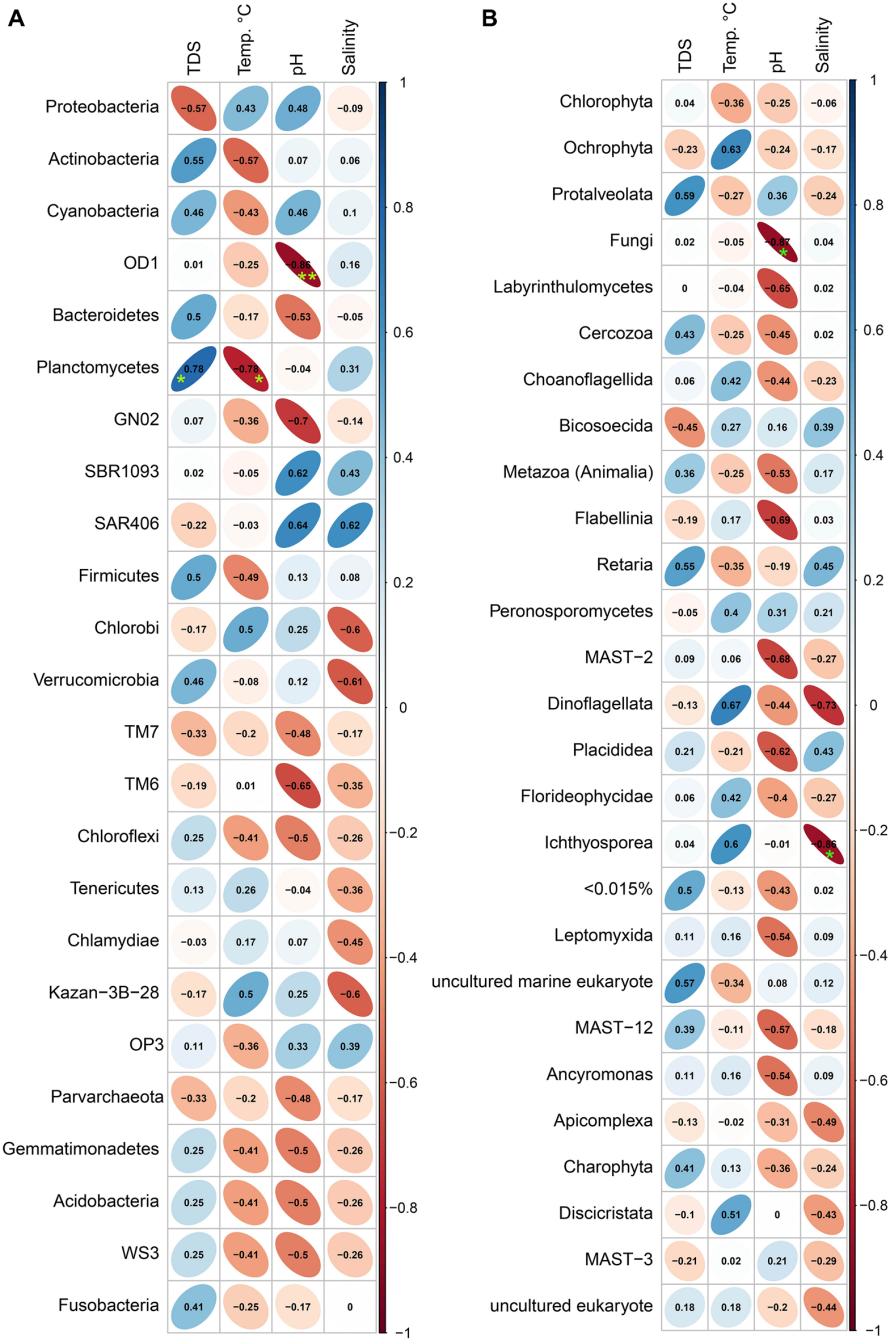
Pairwise Spearman’s correlation of physicochemical parameters and microbial phyla (prokaryotic) and division (eukaryotic) level. (**A**) Correlation with physicochemical parameters (TDS, temperature, pH, and salinity) with 24 phyla of prokaryotes detected in the study areas. (**B**) Correlation with physicochemical parameters with top 26 divisions (> 0.015%) of eukaryotes detected in the study areas. The numbers represent the Spearman’s correlation coefficient (r). Blue and red indicate positive and negative correlations, respectively. The color density, ellipse size, and numbers reflect the scale of correlation. *Significance level (*p < 0.05; **p < 0.01; ***p < 0.001).

The influences of physicochemical factors on the relative abundance of prokaryotic and eukaryotic microbial communities of the samples revealed that Parcubacteria (also known as Candidate Phylum OD1 bacteria (OD1)) showed significant negative correlation with pH (Spearman correlation; r > − 0.86, *p* < 0.01). Planctomycetes demonstrated a substantial positive association with TDS (Spearman correlation; r > 0.78, *p* < 0.01) and a significant negative correlation with temperature (Spearman correlation; r > − 0.78, *p* < 0.01) (Fig. 5A). Fungi and Ichthyosporea showed strong negative correlation with pH (Spearman correlation; r > − 0.86, *p* < 0.01) and salinity (Spearman correlation; r > − 0.87, *p* < 0.01) respectively (Fig. 5B).

### Shotgun metagenomic sequence analysis

#### Taxonomic composition of prokaryotic and eukaryotic microbial community

For assessment of overall community composition and relative functional profiling of surface microbiome of the two coastal regions of BoB, we also performed shotgun metagenomic sequencing using the pooled DNA samples (S1 = Saint Martin; S2 = Cox’s bazar). From the taxonomic profiling data, both S1 and S2 sample showed to harbor bacteria, eukaryotes, archaea and viruses (Supplementary Data-2). Among them, 99.13% and 99.33% sequences revealed presence of bacteria in S1 and S2 respectively, followed by eukaryotes (0.01%, 0.03%), viruses (0.80%, 0.64%) and archaea (0.02%, 0.01%).

**Figure 6:**
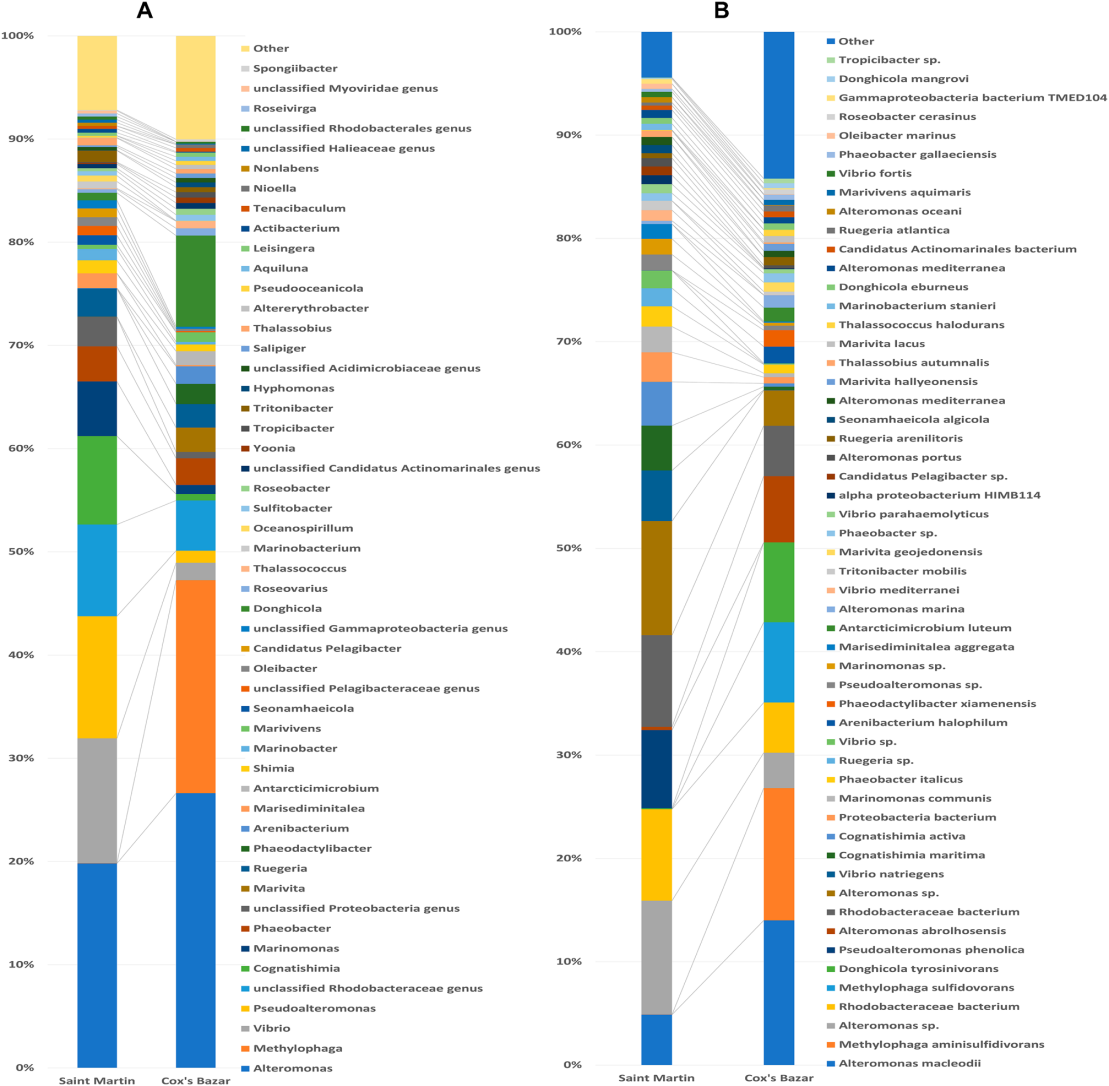
The (**A**) genera and (**B**) species level taxonomic profile of microbes obtained from shotgun metagenomic sequencing of Saint Martin (S1) and Cox’s bazar (S2) samples. Stacked bar plots showing the relative abundance and distribution of the top 50 genus and species. The distribution and relative abundance of the microbes in the study metagenomes are also available in Supplementary Data-2.

*Altermonas* appeared as the most prevalent bacterial genera in both locations, followed by *Methylophaga* for Cox’s Bazar and *Vibrio* for Saint Martin (Fig. 6). Among the other genera *Pseudoalteromonas*, *Rhodobacteraceae*, *Cognatishimia*, *Marinomonas*, *Phaeobacter*, and *Proteobacter* are fairly abundant in from both locations. At the species level, *Alteromonas macleodii* was predominant in both locations followed by *Methylophaga aminisulfidivorans*, *Alteromonas* sp., *Rhodobacteraceae bacterium*, *Methylophaga sulfidovorans*, *Donghicola tyrosinivorans*, *Alteromonas abrolhosensis*, and *Rhodobacteraceae* bacterium for Cox’s Bazar, and *Alteromonas macleodii*, *Methylophaga aminisulfidivorans*, *Alteromonas* sp., *Rhodobacter*aceae bacterium, *Methylophaga sulfidovorans*, *Donghicola tyrosinivorans*, *Pseudoalteromonas phenolica*, *Alteromonas abrolhosensis*, *Rhodobacteraceae bacterium*, *Vibrio natriegens*, *Cognatishimia maritima* and *Cognatishimia active* for Saint Martin.

#### Functional profiling of BoB microbiome

All levels of functional gene profiling using KEGG Orthology (https://www.genome.jp/kegg/ko.html) revealed differential abundance of metabolic genes in two samples. The most abundant metabolic category was the BRITE Hierarchies category (KO09180) present in Cox’s bazar and Saint Martins with a relative abundance of 0.3789 and 0.3826, respectively (Supplementary Figure-3; Suppl. Table-1). Notably, the KEGG Orthology derived functional gene identification showed the presence of human disease-causing genes in the both samples.

The top 15 BRITE level B found in Cox's Bazar and Saint Martin were Protein families involved in signaling and cellular processes (ko09183), genetic information processing (ko09182), amino acid metabolism (ko09105), carbohydrate metabolism (ko09101), metabolism (ko09181), metabolism of cofactors and vitamins (ko09108). It is interesting to note that the distribution of BRITE level B categories is similar between the two locations, with only small differences in the abundance of each category (Supplementary Figure-4; Suppl. Table-1).

In BRITE level C functional gene annotation by KEGG-Orthology revealed that the two sources of marine water samples have similar relative abundances of proteins (Supplementary Figure-5). For example, both locations have relatively high levels of transporters, enzymes with EC (Enzyme Commissioner) numbers, DNA repair and recombination proteins, and transfer RNA biogenesis proteins. There were also some differences between the two sources. Cox's Bazar has higher relative abundances of glycine, serine, and threonine metabolism proteins, as well as porphyrin metabolism proteins, while Saint Martin has higher relative abundances of ABC transporters and peptidases and inhibitors. The most abundant KEGG orthologous group in both locations is K02014 (TC.FEV.OM), which is involved in the transport of amino acids, indicating a higher demand for amino acids in these locations, possibly due to high metabolic activity or protein synthesis. The second most abundant orthologous group in Saint Martin is K03406 (mcp), which is involved in bacterial chemotaxis, whereas in Cox's Bazar K20276 (bapA) is the second highest, which is involved in the formation of biofilms. This suggests that bacterial motility may be important in Saint Martin, while biofilm formation is more important in Cox's Bazar. The major metabolic products and prospects of top 50 genus have been listed in Supplementary Table-2.

**Figure 7:**
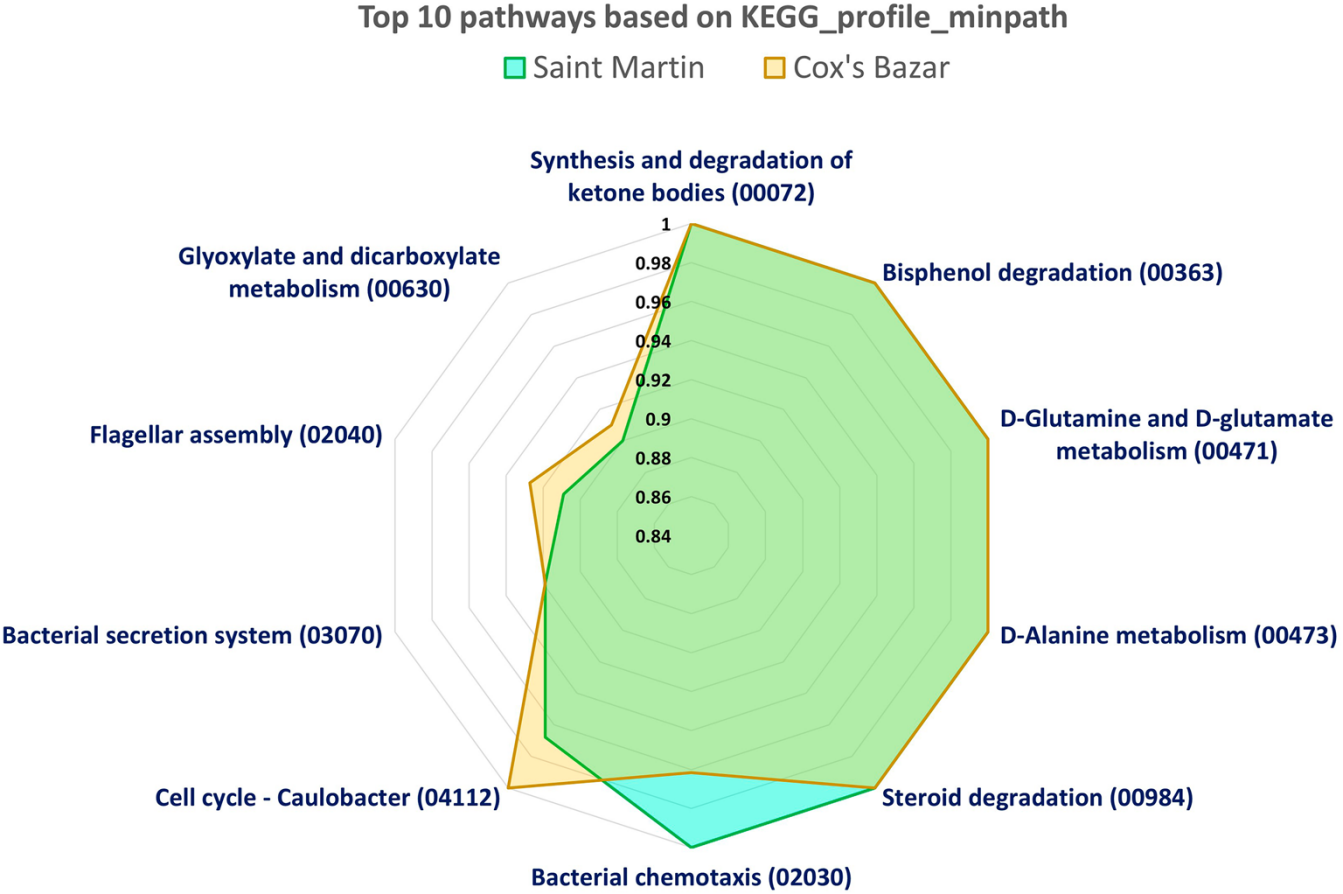
Most abundant (Top 10) pathways present in with the marine microbiome in BoB, Bangladesh (based on KEGG_profile_minpath).

Comparing the relative abundance of metabolome of two samples, a significant difference in biodegradation metabolism of the two microbiomes have been revealed. Figure 7 illustrates the relative abundance of top 10 metabolic genes prevalent in the functional microbiome of two samples, determined from shotgun metagenome sequences of S1 and S2 (Fig. 7). Importantly, the relative abundance of Bis-phenol degradation metabolism is higher in both samples, indicating the presence of potential microbial communities capable for possible photodegradation of bisphenol-A (BPA) which is a harmful component found in hard plastics, water bottles etc. The abundance of D-Glutamate and D-Glutamine metabolism indicates the continuous fixation of atmospheric nitrogen by the marine bacteria and anabolic utilization of these amino acids for biosynthesis of proteins, nucleic acids in microorganisms.

#### Antibiotics resistance gene families prevalent in coastal water microbiome of Saint Martin and Cox’s Bazar

In total, 54 antimicrobial and metal resistance genes (Supplementary Tables 3, 4) were detected in the coastal water samples from BoB considering the gene coverage above 80%. Among them, 17 and 48 genes belong to S1 and S2, respectively. Only 11 antimicrobial resistance (AMR) genes were found in both samples, whereas 6 and 37 genes were unique to S1 and S2 sample respectively. Saint Martin (S1) sample had relatively a smaller number of resistance genes where macrolide-resistance being the most abundant one, followed by aminoglycoside-resistance and quinolone-resistance. On the other hand, Cox’s Bazar (S2) samples had nearly three times more resistance than S1 samples with phenicol resistance gene being the most abundant one, followed by resistance to tetracycline, quinolone, macrolide and sulfonamide. Cox’s Bazar samples also encoded genes for resistance to various biocides and metals (Table 1). No resistance genes for tetracycline, phenicol and sulfonamides with > 80% gene coverage have been found in S1 samples. Likewise, resistance genes for trimethoprim (with > 80% coverage) have not been identified in S2 samples.

**Table 1:** Antimicrobial resistance gene profiling for S1 and S2 samples.

Antimicrobial class	Resistance genes (> 80% coverage)		Major mode of resistance
	S1 samples	S2 samples	
Aminoglycosides	A16S group *rrs*C, *rrs*H, *rps*L,	*aadA1, aac*(6')-*Ib11, aac*(6')-Ib, A16S group, *rrs*C, *rrs*H	Aminoglycoside N-acetyl transferase; Aminoglycoside-resistent 16 s ribosomal subunit protein
Tetracycline		*tet(*G), *tet*(X)	Tetracycline efflux MFS transporter Tet(G) Tetracycline-inactivating monooxygenase Tet(X)
Fluoroquinolones and Quinolones	*qnr*VC *gyr*A	*qnr*S	quinolone resistance pentapeptide repeat protein QnrVC1 and QnrVC4 quinolone resistance pentapeptide repeat protein QnrS2 Fluoroquinolone-resistant DNA topoisomerases
Phenicols		*flo*R2 *cat*B *flo*R	Chloramphenicol/florfenicol efflux MFS transporter FloR Chloramphenicol_acetyltransferases Phenicol_resistance_MFS_efflux_pumps
Macrolide	*erm (MLS23s group)*	*ere*(A) *mph*(F) *erm*(F) *ere*(D) *mph*E MLS23S Group	EreA family erythromycin esterase Mph(F) family macrolide 2'-phosphotransferase 23S rRNA (adenine(2058)-N(6))-methyltransferase Erm(F) EreD family erythromycin esterase Macrolide_phosphotransferases Macrolide resistant 23SrRNA mutation
Sulfonamides		*sul*1 *sul*2	Sulfonamide-resistant dihydropteroate synthase Sul1 Sulfonamide-resistant dihydropteroate synthase Sul2
Trimethoprim	*dfr*A6		Trimethoprim-resistant dihydrofolate reductase DfrA6
Elfamycins	TUFAB group	TUFAB group	EF-Tu Inhibition
Metal resistance		*mer*C, *mer*T *mer*R1	Mercury_resistance_protein Mercury_resistance_regulator
Biocide and Quaternary Ammonium Compounds	*vme*Z *vme*D	*qac*E QACEDELTA1	Multi-biocide RND efflux pump Quaternary ammonium compound efflux SMR transporter QacE Drug_and_biocide_SMR_efflux_pumps
Cationic antimicrobial peptides	CAP16S group	CAP16S group	Cationic peptide-resistant 16S ribosomal subunit protein

#### Gene families associated with virulence factors and Mobile genetic elements revealed from shotgun metagenome of BoB

From the analysis of functional properties of the prevalent microbiome of BoB, several genes related to virulence factors have been identified. The EzBioCloud and AMR++pipelines both identified bacterial pathogenic genes mostly related to flagellar motility, such as *flgB, flgC, flgD, mshA, fliA* etc. (Supplementary Table-5). Other genes for chemotaxis (*cheY*), transport protein (*pyuC, pysC*) and type II secreteion system protein (*epsE, epsG*) have been identified, which are involved in flagellar motility, nutritional uptake of metal Fe-like metal ions and secretion of effector moieties for flagella formation. Interestingly, most of the virulence genes identified from S1 sample had gene coverage > 80%, whereas no genes from S2 samples had above 80%. Regardless of the coverage, shotgun metagenome sequence analysis of both samples has been determined to have significant presence of virulence genes which indicate that the coastal water of both locations is harboring pathogenic organisms. Notably, taxonomic identifications revealed presence of a number of pathogenic bacteria in the samples, justifying the source of virulence genes. Both the S1 and S2 shotgun sequences have been analyzed to detect mobile genetic elements, which revealed the prevalence a number of diverse transposon gene families, like Tn3, Tn402, Tn554, Tn7 etc. Besides, a number of IS elements and Integron gene families have been identified in both S1 and S2 sample. 11 and 41 complete sequences of plasmids have been identified in S1 and S2 respectively (Supplementary Data-2). The proportion of mobile genetic elements and plasmids in two shot sequence data has been illustrated in Supplementary Figure-6. The plasmids may play vital role in dissemination of antimicrobial resistance genes, virulome and other non-marine genetic moieties to the coastal microbiome.

## Discussion

Coastal microbiome research, particularly in the context of Bangladesh’s south and south-east coast, is still in its infancy. As a part of the Indian Ocean, the third largest oceanic division of the world^83^, and being surrounded by three different countries, the BoB provides ecological habitats and niches for an enormous diversity of microbial groups^13,84^. Notably, Bangladesh has the longest sea-beach in the world and the south border of the country is completely on the shore of Bay of Bengal, including the Sundarban, largest mangrove forest. The geographical location and surrounding shoreline ecosystem of BoB are of immense importance for understanding the indigenous microbial community, impact of climate changes on the biosphere of BoB and spatiotemporal dysbiosis of the BoB ecosystem. In this study, we sought to expand the microbiome research in this understudied area by investigating the microbial profile of two coastal sites in Bangladesh. The two sites were only around 50 nautical miles away from each other and didn’t significantly vary in environmental physiochemical parameters or microbial community composition. The microbial profiling conducted in this study was produced using “universal” PCR primers, selected for their ability to simultaneously target both 16S and 18S rRNA genes. Microbial communities are now well understood as major contributors in maintaining balance in marine and terrestrial ecosystems and understanding the baseline ambient-conditioned microbiome can help future efforts to monitor shifts in microbiome responses to both short- and long-duration environment changes through processes including physiological acclimation, compositional shifts, and evolution.

The 16S rDNA based microbial profiling conducted in this study revealed high bacterial diversity in the coastal regions of Cox's Bazar and Saint Martin with many different bacterial taxa being represented. Overall, the surface aquatic community was dominated by the Rhodobacteriaceae family, which are the major group of microorganisms involved in organic matter recycling in marine environments^85^. The Rhodobacteraceae family has been identified by 49 OTUs, a large number of which were classified to the genus level. The notable genera of Rhodobacteraceae are *Nautella, Anaerospora, Antarctobacter, Thalassobius, Thalassococcus, Roseivivax,* and *Roseovarius*. The Rhodobacteraceae family of bacteria typically flourish in marine settings and they mostly consist of aerobic photo- and chemoheterotrophs that are involved in symbiosis as well as contributors to sulfur and carbon biogeochemical cycles^85^. The second most abundant family, the Flavobacteriaceae, have been identified by 45 different OTUs s, many of which were identified to the genus level. According to a previously published report, in the maritime environment, members of the bacterial family Flavobacteriaceae are extensively dispersed and frequently discovered in association with algae, fish, debris, or marine animals^86,87^. The ability of marine Flavobacteriaceae to consume a variety of carbon sources is supported by the high frequency and diversity of genes encoding polymer-degrading enzymes, which are frequently organized in polysaccharide utilization loci (PULs)^88,89^. With a high incidence of gene clusters encoding pathways for the generation of antibiotic, antioxidant, and cytotoxic chemicals, Flavobacteriaceae have a varied arsenal of secondary metabolite biosynthesis^89^. Relatively higher abundance of the Flavobacteriaceae family in our study sites could indicate the availability of complex macromolecules in these coastal regions. These findings in the BoB confirm previous studies that showed the dominance of Proteobacteria and Bacteroidetes, of which Flavobacteriaceae and Rhodobacteraceae are a part of, in multiple other locations within the bay^13,90^.

From the sample-wide analysis of 16S data (Fig. 4A), there were notable abundances of *Pseudoalteromonas, Alteromonas* and *Methylophaga* genus in S6, S9 and S12 respectively. *Pseudoalteromonas* species exhibit antibacterial, bacteriolytic, agarolytic, and algicidal properties and are typically found associated with marine eukaryotes^91,92^. Additionally, several isolates of *Pseudoalteromonas* stop the growth of typical fouling species. The genus *Alteromonas* have a wide range of habitats, including coastal and open ocean regions, deep sea and hydrothermal vents, and marine sediments^93^. *Alteromonas* is also known to have a wide variety of metabolic activities, including the breakdown of complex organic molecules^94^.

Among the other prominent genera found at both sites, *Anaerospora*, *Marivita,* and *Vibrio* were identified, with *Vibrio* being of particular interest due to its several potentially pathogenic species^95^. The presence of these bacteria in Cox's Bazar water sample suggests that careful monitoring of their populations may be required to prevent potential negative impacts on human and animal health. The genus *Marinomonas*, which have been detected only in Jetty samples (S8), is considered as a promising candidate for potential biotechnological applications, such as the production of enzymes, biofuels, and biodegradable plastics^96–98^.

The vast majority of eukaryotic OTUs from Cox's Bazar (74.27%) and Saint Martin (88.85%) could not be assigned to any recognized divisions. Since there is large variability in the targeted 18S rRNA gene, amplification-based molecular methods can be problematic for eukaryotic organisms^99^. To address this issue some studies utilized a chloroplast 16S rRNA gene database for taxonomic assignments of photosynthetic eukaryotic organisms^14^. For our study we sequenced the V9 region of 18S rRNA which has been shown to have a higher resolution at the genus level (80% identification rate)^100^. However, genomic data from this part of BoB is very limited—therefore, the existing databases might have lower resolution in assigning the taxonomic profiles. Including other regions of the 18S rRNA, i.e., V2 and V4 might have recovered higher diversity of microbial eukaryotes in these regions. Nonetheless, many eukaryotes were able to be identified with *Paraphysomonas,* being the most widespread and abundant.

Marine microorganisms exhibit numerous metabolic capabilities either as independent strains or as members of complex microbial consortia. They can produce eco-friendly chemicals and novel metabolites that can be used in the management and treatment of environmental waste, such as nontoxic biosurfactants and biopolymers and for the treatment of diseases^101–104^. Many of the microbial lineages previously reported to synthesize antibiotic compounds have also been discovered in our study sites (Supplementary Table-2). These include *Rhodobacteraceae bacterium*^105^, *Pseudoalteromonas phenolica*^106^, *Proteobacteria bacterium*^107^, *Ruegeria* sp.^108^, *Vibrio mediterranei*^109^, *Phaeobacter* sp.^110^ and *Marinomonas ostreistagni*^111^ among others*.* Other microorganisms like *Alteromonas portus*^112,113^ and *Seonamhaeicola algicola*^114,115^ are known for production of antioxidants carotenoids, zeaxanthin; *Alteromonas oceani*^116^ and *Ruegeria* sp.^108^ for probiotics; *Alteromonas portus*^117^ for anticancer activity; *Vibrio fortis* for biofouling^118,119^ and *Phaeobacter italicus* for biodiesel prospects^117,120^.

Bangladesh has an extreme shortage of facilities and infrastructures for treatment of hospitals and municipal waste^121,122^. In fact, most wastes are disposed into the freshwater bodies, like rivers, canals, lakes etc., which eventually reach the estuarine and marine waters of the Bay of Bengal. This substantial agricultural runoff, as well as anthropogenic hospital and municipal discharge cause deposition of antibiotics and ARB in the surrounding coastal environment^121^. Antimicrobial resistance (AMR) genes and residual antibiotics potentially impact the overall community composition and eventually threatening the ecological balance of microorganisms through unwanted exposure of autochthonous microbial community to the antimicrobial compounds and hereby disturbing the harmony of ecosystem health. It has already been documented that when naturally untainted environments are contaminated by ARB and ARGs, they can mobilize ARGs to naive bacterial communities^123,124^. Although many studies have investigated the metabolic potential of the marine microbes in other oceanic regions, the functional and phylogenetic diversity of the microbial community in the coastal water of the BoB remain underexplored.

Our in-depth metagenomic analysis revealed presence of antibiotic resistance genes in multiple classes (Supplementary Tables 3 and 4) in the coastal microbial community of Saint-martin (S1) and Cox’s bazar (S2). Saint Martin Island microbial community harbored resistance genes against macrolides, aminoglycosides, and quinolones. On the other hand, the Cox’s bazar microbes contained larger spectrum of AMR genes, with higher coverage and abundance of each gene. These findings indicate the presence of antibiotic resistance genes in the surface waters of BoB, with higher abundance in the Cox’s Bazar region. As this area is highly inundated with tourists year-round, the coastal water encounters microbial populations originated from human and animals, allowing an intrusion and environmental adaptation of the allochthonous microbes into the natural microbial community. Besides, wastes from the coastal districts, including the second largest and populated city of Bangladesh “Chattogram”, are being dumped and carried away to the marine water through all the rivers connected to the BoB^122,125,126^. Discharged waste coming from hospital and municipal sources contain reservoirs of antibiotics which are harbored in the feces of humans, chickens, and cows. Resistance against colistin-like last-resort antibiotics have been reported to be disseminated into the microbiome of marine water^127^, although this was not found in the samples we studied. The resistomes of BoB microbiome strongly exemplifies how anthropogenic input can turn the coastal environment into a potential reservoir of antibiotic resistance, further threatening the public health. Additionally, pathogens causing food borne illnesses like *Vibrio parahaemolyticus* were also found. Given the implications for public health and marine ecological balance, future studies on the BoB coast as a potential sink and source of antibiotic resistance will be crucial.

While our study provided a baseline profiling of the bacterial and microeukaryote communities in the surface waters in the BoB, there were many limitations in our approach. Our sampling methodology likely allowed for higher proportion of planktonic bacteria to be captured—as we passed the water samples through filters of pore size 11 µm, which excluded some nanoplanktons (2–20 µm) and all microplanktons (20–200 µm). In addition, it might have also excluded microbial communities in association with particles and/or forming biofilms. Subsequently, the samples were passed through 0.45 µm followed by 0.22 µm membranes. The later approach removed many of the Femtoplanktons (0.01–0.2 µm) i.e., viruses. Therefore, only the cell associated viruses and viruses larger than 0.2 µm were contained in the membranes, making our samples contain mostly the picoplankton (0.2–2 µm) such as bacteria, small eukaryotes, archaea, and some viruses. Additionally, deeper sequencing and higher sample volume would potentially lead to a better estimate of the microbial diversity in our samples. Regardless of these limitations, our shotgun, 16S and 18S metagenomic sequencing revealed presence of at least 60 different phyla, total of 397 prokaryotic OTUs representing 24 bacterial phyla and one archaeal phylum, and 693 OTUs for eukaryotes representing 44 divisions.

Overall, this work purported to survey and describe the surface water microbial communities in the understudied waters of the BoB, Bangladesh. This work lays the foundation for future study into this region as it could be seen as a reservoir for both helpful bacterial metabolites as well as potential pathogens and resistant strains. Many open questions currently limit our capacity to assess how microbial processes influence the ecology of these environments, both under contemporary conditions and under future environmental change. Therefore, there is a clear need to prioritize and define key questions for future research that will allow for better assessments of how microbial processes truly influence the ecology and health of coastal marine environments.

## Conclusion

The findings from this study provide the first insights into the properties, taxonomic composition and functional profiles of coastal microbial communities of the Bay of Bengal from Bangladesh. Our combined approach for 16S and 18S amplicon-based sequencing provides a much more comprehensive picture of the sublittoral epipelagic coastal microbiome of BoB. The shotgun metagenomic analysis of these microbiomes reveals significantly abundant communities and their functional potential. This debuting microbial community profiles can be the potential baseline database for future studies focusing the aquatic microbiome of coastal area with very low anthropogenic footprints, the climate-change impacts and the comparative analysis of coastal and deep-sea metagenomes to explore the bio-prospective potential of the Bay of Bengal.

### Supplementary Information

Supplementary Information 1.

Supplementary Information 2.

Supplementary Figures.

Supplementary Tables.

## Data Availability

The 16S, 18S and Shotgun sequences are available in BioProject PRJNA936421, PRJNA936461 and PRJNA936489, respectively of NCBI database. All supplementary files are uploaded along with the manuscript.
