# Author Correction: Phylogenetic diversity and functional potential of the microbial communities along the Bay of Bengal coast

**DOI:** 10.1038/s41598-023-45996-2

**Published:** 2023-11-01

**Authors:** Salma Akter, M. Shaminur Rahman, Hazrat Ali, Benjamin Minch, Kaniz Mehzabin, Md. Moradul Siddique, Syed Md. Galib, Farida Yesmin, Nafisa Azmuda, Nihad Adnan, Nur A. Hasan, Sabita Rezwana Rahman, Mohammad Moniruzzaman, Md Firoz Ahmed

**Affiliations:** 1 Department of Microbiology, Jahangirnagar University, Savar, Dhaka, Bangladesh; 2 Department of Microbiology, Jashore University of Science and Technology, Jashore, Bangladesh; 3 Department of Marine Biology and Ecology, Rosenstiel School of Marine, Atmospheric, and Earth Science, University of Miami, Coral Gables, FL USA; 4 Department of Computer Science and Engineering, Jashore University of Science and Technology, Jashore, Bangladesh; 5 University of Maryland, College Park, MD USA; 6 Department of Microbiology, University of Dhaka, Dhaka, Bangladesh

Correction to: *Scientific Reports* 10.1038/s41598-023-43306-4, published online 25 September 2023

The original version of this article contained errors in the legends of Figures 1, 2, and 3.

Figure 1. Sampling location and their physicochemical properties. (**A**) Two sampling locations (Cox’s Bazar and Saint Martin) are indicated (yellow rectangle). (**B**) The physicochemical parameters (pH, salinity, TDS and Temperature) of each are plotted on boxplots and comparisons were made with *t*-test. The map was constructed using ArcGIS online platform.

Now reads:

Figure 1. Sampling location and their physicochemical properties. (**A**) Two sampling locations (Cox’s Bazar and Saint Martin) are indicated. (**B**) The physicochemical parameters (pH, salinity, TDS and Temperature) of each are plotted on boxplots and comparisons were made with t-test. The map was constructed using ArcGIS online platform.

Figure 2. Prokaryotic and eukaryotic microbial alpha- and beta-diversity based on 16S and 18S taxonomic abundance. (**A**) For the prokaryotic (bacteria and archaea) microbial community of Cox's Bazar and Saint Martin samples, the observed species, Chao1, Shannon, Simpson, InvSimpson, and Fisher diversity (Alpha diversity) indices were estimated. X-axis represents the location and y-axis represents the alpha diversity measure. The diversity for each is plotted using boxplots, and the pairwise Wilcoxon sum rank test is used to compare them. (**B–G**) Beta diversity measures of the prokaryotic (bacteria and archaea) microbial community. Principal coordinate analysis (PCoA) (**B–D**) and non-metric multidimensional scaling (**E–G**) were performed using Bray, Weighted-Unifrac, and Unweighted-Unifrac distance metrics for the two locations of samples. Permutational multivariate analysis of variance (PERMANOVA) was performed with 999 permutations to estimate a significance (*p* value) for differences between two locations. PERMANOVA with 999 permutations was used to determine the significance (*p* value) of differences between two locations. Significance level (*p* value) 0.0001, 0.001, 0.01, 0.05, and 0.1 are represented by the symbols "****", "***", "**", "*", and “n.s”, respectively. Stress value represents the goodness of fit of NMDS (> 0.2 Poor, 0.1–0.2, Fair, 0.05–0.1 Good, and < 0.05 Excellent). (**H**) Comparison of relative abundance of twenty-five prokaryotic phyla and (**I**) Genus in the two different locations (Cox’s Bazar and Saint Martin). The diversity for each division is plotted on boxplots and comparisons are made with Wilcoxon sum rank test. Significance level (*p* value) 0.0001, 0.001, 0.01, 0.05, and 0.1 are represented by the symbols “****”, “***”, “**”, “*”, and “n.s”, respectively.

Now reads:

Figure 2. Bacterial and Archaeal alpha- and beta-diversity and taxonomic abundance based on 16S amplicon sequencing data. ( A ) For the prokaryotic (bacteria and archaea) microbial community of Cox's Bazar and Saint Martin samples, the observed species, Chao1, Shannon, Simpson, InvSimpson, and Fisher diversity (Alpha diversity) indices were estimated. X-axis represents the location and y-axis represents the alpha diversity measure. The diversity for each is plotted using boxplots, and the pairwise Wilcoxon sum rank test is used to compare them. (B–G ) Beta diversity measures of the prokaryotic (bacteria and archaea) microbial community. Principal coordinate analysis (PCoA) ( B–D ) and non-metric multidimensional scaling ( E–G ) were performed using Bray, Weighted-Unifrac, and Unweighted-Unifrac distance metrics for the two locations of samples. Permutational multivariate analysis of variance (PERMANOVA) was performed with 999 permutations to estimate a significance ( p -value) for differences between two locations. PERMANOVA with 999 permutations was used to determine the significance ( p -value) of differences between two locations. Significance level ( *p* -value) 0.0001, 0.001, 0.01, 0.05, and 0.1 are represented by the symbols "****", "***", "**", "*", and "n.s", respectively. Stress value represents the goodness of fit of NMDS (> 0.2 Poor, 0.1–0.2, Fair, 0.05–0.1 Good, and < 0.05 Excellent). ( H ) Comparison of relative abundance of twenty-five prokaryotic phyla and ( I ) Genus in the two different locations (Cox’s Bazar and Saint Martin). The diversity for each division is plotted on boxplots and comparisons are made with Wilcoxon sum rank test. Significance level (*p* -value) 0.0001, 0.001, 0.01, 0.05, and 0.1 are represented by the symbols "****", "***", "**", "*", and "n.s", respectively.

Figure 3. (**A**) The observed species, Chao1, Shannon, Simpson, InvSimpson, and Fisher diversity (Alpha diversity) measures were used to estimate the Eukaryotic microbial community diversity of Cox's Bazar and Saint Martin samples as described for the prokaryotic microbes. (**B–G**) Beta diversity of the eukaryotic microbial community was estimated here as described in Figure 2B–G. For the two sample sites, Bray, Weighted-Unifrac, and Unweighted-Unifrac distance measures were used. Permutational multivariate analysis of variance (PERMANOVA) was performed with 999 permutations to estimate a significance (*p* value) for differences between two locations. PERMANOVA with 999 permutations was used to determine the significance (*p* value) of differences between two locations. Significance level (*p* value) 0.0001, 0.001, 0.01, 0.05, and 0.1 are represented by the symbols "****", "***", "**", "*", and "n.s", respectively. Stress value represents the goodness of fit of NMDS (> 0.2 Poor, 0.1–0.2, Fair, 0.05–0.1 Good, and < 0.05 Excellent). (**H**) Comparison of relative abundance of twenty-five eukaryotic divisions and (**I**) Order in the two different locations (Cox’s Bazar and Saint Martin). The diversity for each order is plotted and differences were tested using Wilcoxon sum rank test. Significance level (*p* value) 0.0001, 0.001, 0.01, 0.05, and 0.1 are represented by the symbols “****”, “***”, “**”, “*”, and “n.s”, respectively.

Now reads:

Figure 3 Eukaryotic microbial diversity and taxonomic abundance based on 18S amplicon sequencing data. (**A**) The observed species, Chao1, Shannon, Simpson, InvSimpson, and Fisher diversity (Alpha diversity) measures were used to estimate the Eukaryotic microbial community diversity of Cox's Bazar and Saint Martin samples. (**B–G**) Beta diversity of the eukaryotic microbial community was estimated here as described in Figure-2. For the two sample sites, Bray, Weighted-Unifrac, and Unweighted-Unifrac distance measures were used. Permutational multivariate analysis of variance (PERMANOVA) was performed with 999 permutations to estimate a significance (*p*-value) for differences between two locations. PERMANOVA with 999 permutations was used to determine the significance (*p*-value) of differences between two locations. Significance level (*p*-value) 0.0001, 0.001, 0.01, 0.05, and 0.1 are represented by the symbols "****", "***", "**", "*", and "n.s", respectively. Stress value represents the goodness of fit of NMDS (> 0.2 Poor, 0.1–0.2, Fair, 0.05–0.1 Good, and < 0.05 Excellent). (**H**) Comparison of relative abundance of twenty-five eukaryotic divisions and (**I**) Order in the two different locations (Cox’s Bazar and Saint Martin). The diversity for each division is plotted and differences were tested using Wilcoxon sum rank test. Significance level (*p*-value) 0.0001, 0.001, 0.01, 0.05, and 0.1 are represented by the symbols "****", "***", "**", "*", and "n.s", respectively.

The original article has been corrected.

