# Supplementary material for: Phylogenetic diversity and functional potential of the microbial communities along the Bay of Bengal coast
Source: Sci Rep. 2023 Sep 25;13:15976. doi: 10.1038/s41598-023-43306-4 (PMC10520010; doi:10.1038/s41598-023-43306-4)
Supplement: Supplementary file 3 — Supplementary Figures. [file 41598_2023_43306_MOESM3_ESM.docx]

**Supplementary Figures**


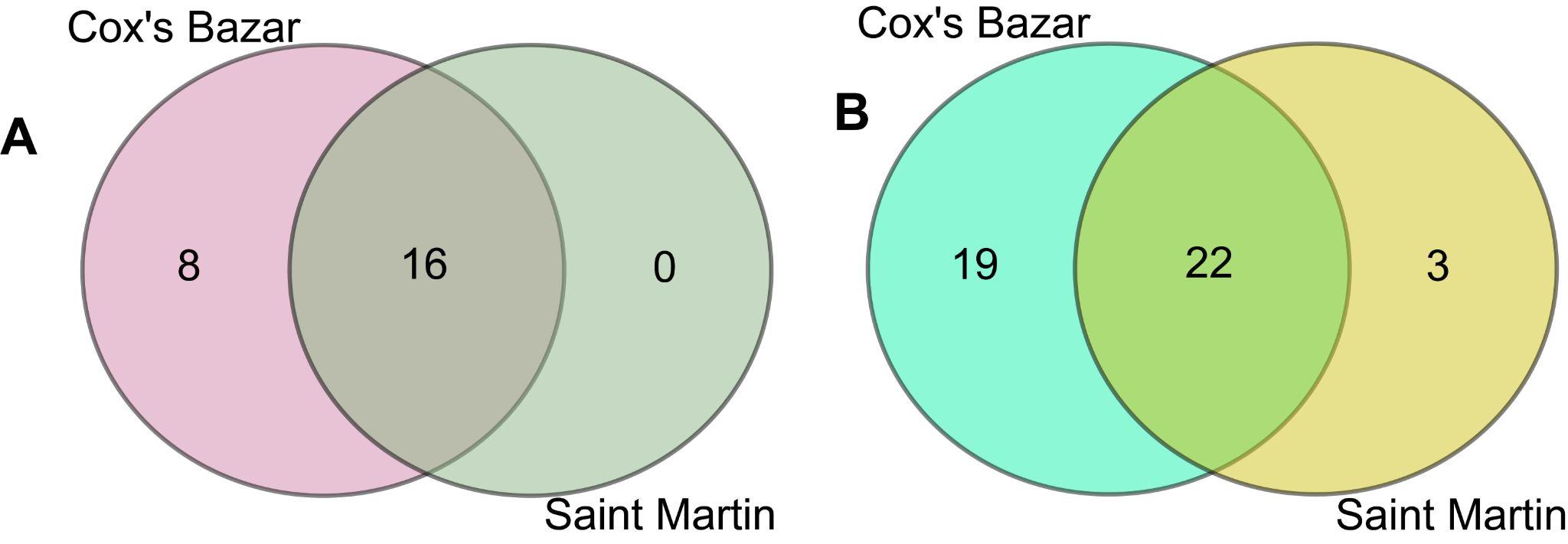
 **Figure-1: Venn diagram of phylum and division level overlap of prokaryotic (A) and eukaryotic (B) samples from two locations.** Venn diagrams show common and unique microbial populations in the sampling locations.

**Figure-2: Comparison of relative abundance of forty-nine prokaryotic genus in the two different locations (Cox’s Bazar and Saint Martin).** The diversity for each genus is plotted on boxplots and comparisons are made with Wilcoxon sum rank test. Significance level (p-value) 0.0001, 0.001, 0.01, 0.05, and 0.1 are represented by the symbols "****", "***", "**", "*", and "n.s", respectively.

**Figure-3: Brite Hierarchies’ Level-A gene abundance in metagenome data of Cox’s Bazar (S2) and Saint Martin (S1). No significant differences in Level-A metabolic profile between the samples.**

**Figure-4: Top 15 Brite Hierarchies in Level B (top 15 genes) showing similar abundance of metabolic genes in microbiome of Saint Martin and Cox’s Bazar.**

**Figure-5: Top 15** **Brite Hierarchies in Level C (top 15 genes) showing abundance of Porphyrin metabolism in Saint Martin samples (S1).**


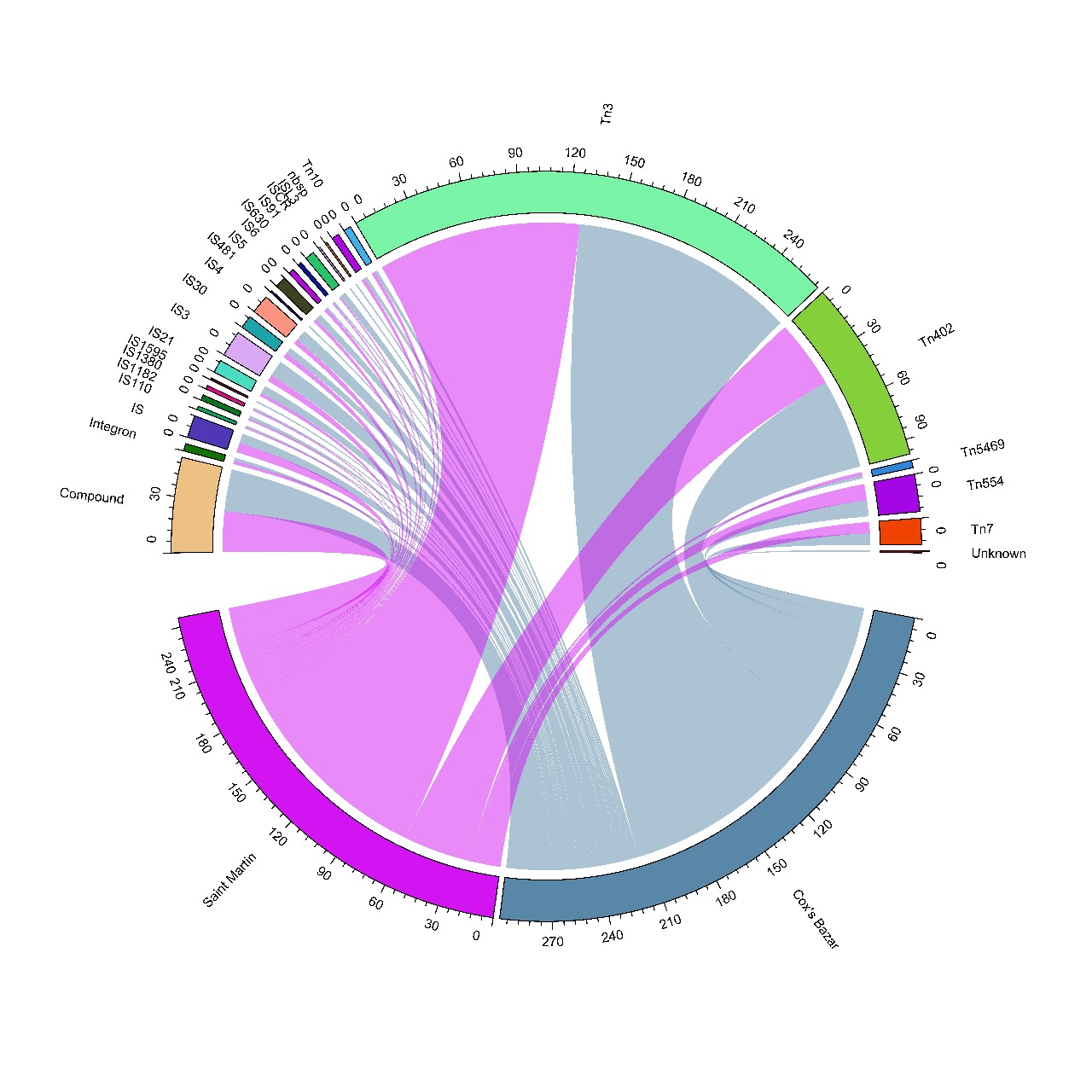


**Figure-6: Relative abundances of mobile genetic elements in Saint Martin and Cox’s Bazar. Tn3 family of transposon are present in highest abundance in both samples.**
